# Rush Med. College

**Published:** 1875-02

**Authors:** Henry L. Harrington


### RUSH MEDICAL COLLEGE.

*Clinic by* PROF. GUNN. *December* 31, 1874.

(Reported by HENRY L. HARRINGTON.)

CASE — Fracture of the Os Innominatum at the bottom of the Acetabulum.

Son of R. J. Fowler; aged 11. The patient, a well developed, robust lad, comes from Storm Lake, Iowa, for advice, giving the following history:

Thirteen days ago he was thrown from a horse in a frozen ploughed field; when picked up he was found lying with his right hip upon a large frozen clod.

A physician was called, who pronounced it a case of fracture of the ala of the right ilium and treated accordingly. Eight days afterwards, the father, suspecting that all was not right, called in consultation other physicians, who decided that the head of the femur was dislocated into the thyroid foramen: accordingly strenuous efforts were made at reduction, but without avail. The patient was able at all times to move the injured limb, and walked about the house the fifth day after the injury. He did not at any time suffer from fever, nor from any other symptoms of constitutional disturbance.

On examination, we notice when the patient is walking, a decided halt in his gait. When standing, we observe marked flattening of the right trochanteric region; great apparent lengthening of the right leg; slight eversion of the right foot; and also that his body is strongly inclined towards the right side, and slightly bowed forwards. At this stage of the examination I suspect that we have downward and forward dislocation of the femur. Upon placing the patient in the supine position and searching carefully in the groin for the head of the bone we fail to find it; we also find that the body and legs are straight, and that although the foot is everted, it can be rotated inwards voluntarily co-extensive with the sound side. Upon careful measurement we find that the elongation is *only* apparent, and due to the inclination of the pelvis. Again, upon putting the leg through its motions, we notice, that with the exception of extreme flexion and extension, they are as perfect and as devoid of pain as those of the left limb. We will now be obliged to give up entirely the idea of dislocation.

There is only one positive sign left upon which to base a diagnosis, *i. e.*, the flattening of the trochanteric region. This is so great, that a straight edge placed along the outer side of the injured side, rests upon the external malleolus, the great trochanter and the crest of the ilium, while upon the sound side it rests upon the malleolus and great trochanter only, being separated from the crest of the ilium at least one and one-fourth inches. Can it be an impacted fracture of the neck? I think not, from the fact that it would not account for the degree of flattening present; for the reason that in that injury we usually have some shortening, and also because the efforts at reduction would probably have separated the fragments. We must, therefore, look to the inner extremity for the injury. I do not think it can be impaction of the neck within the head, because that would not account for the flattening. I am now of the opinion that we have a fracture at the bottom of the acetabulum, with bulging inwards of the fragments, which allows the head of the femur to occupy a plane nearer the mesian line of the body. To confirm or to refute this diagnosis, the patient was placed under the influence of ether, and an examination made per rectum, which revealed a prominent tumor projecting into the pelvic cavity, corresponding in position to the right acetabulum.

It is a remarkable case, from the fact that we have had no symptoms of inflammation of the pelvic organs or tissues. No treatment is required; the lad will undoubtedly regain very good use of the leg.

